# Supplementary material for: Epidemiological inference for emerging viruses using segregating sites
Source: Nat Commun. 2023 May 29;14:3105. doi: 10.1038/s41467-023-38809-7 (PMC10226718; doi:10.1038/s41467-023-38809-7)
Supplement: Supplementary file 1 — Supplementary Information [file 41467_2023_38809_MOESM1_ESM.pdf]

Epidemiological inference for emerging viruses using segregating sites

Supplementary Information

Authors: Yeongseon Park[1], Michael Martin[1,4], Katia Koelle[2,3,*]

[1] Graduate Program in Population Biology, Ecology, and Evolution, Emory University, Atlanta, GA 30322, USA

[2] Department of Biology, Emory University, Atlanta, GA 30322, USA

[3] Emory Center of Excellence for Influenza Research and Response (CEIRR), Atlanta GA, USA

Corresponding author *: katia.koelle@emory.edu

Present affiliation [4]: Department of Pathology, Johns Hopkins University School of Medicine, Baltimore, Maryland, USA

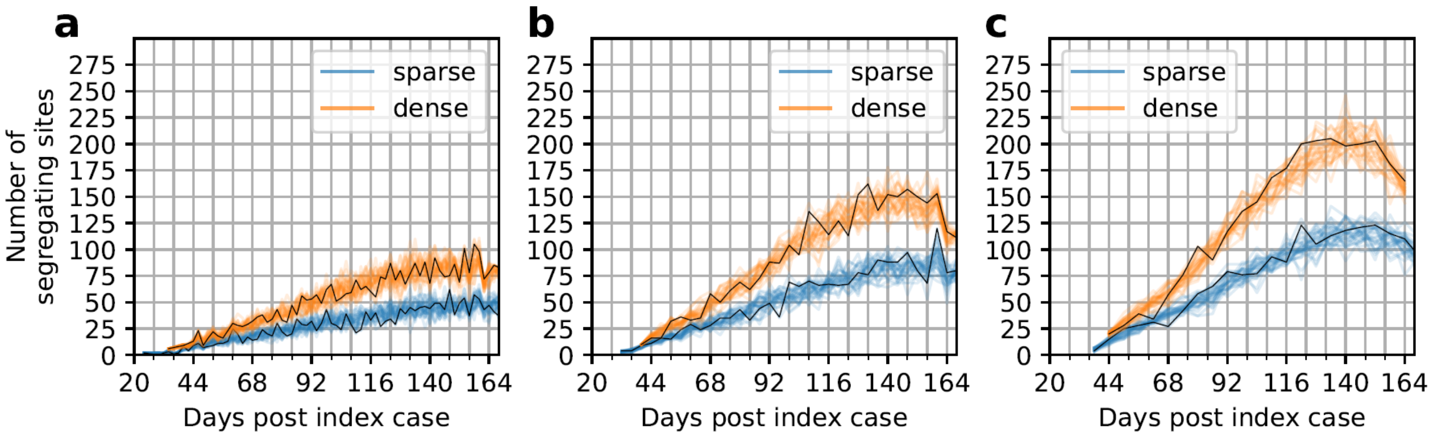

Figure S1. Segregating site trajectories under different time window lengths. Segregating site trajectories for the simulation shown in Figure 1a under dense (orange) and sparse (blue) sampling effort, when trajectories are calculated using time window lengths of (a) 2 days; (b) 4 days (as in Figure 1b); and (c) 6 days. Under the dense sampling scheme, sampling effort is 20 sequences per 2 day time window (a), 40 sequences per 4 day time window (b), and 60 sequences per 6 day time window (c). Under the sparse sampling scheme, sampling effort is 10 sequences per 2 day time window (a), 20 sequences per 4 day time window (b), and 30 sequences per 6 day time window (c). 30 randomly-sampled segregating site trajectories are shown for each sampling effort. Black lines each show a single representative segregating site trajectory. These lines are included to highlight the extent of sampling noise present under different window sizes.

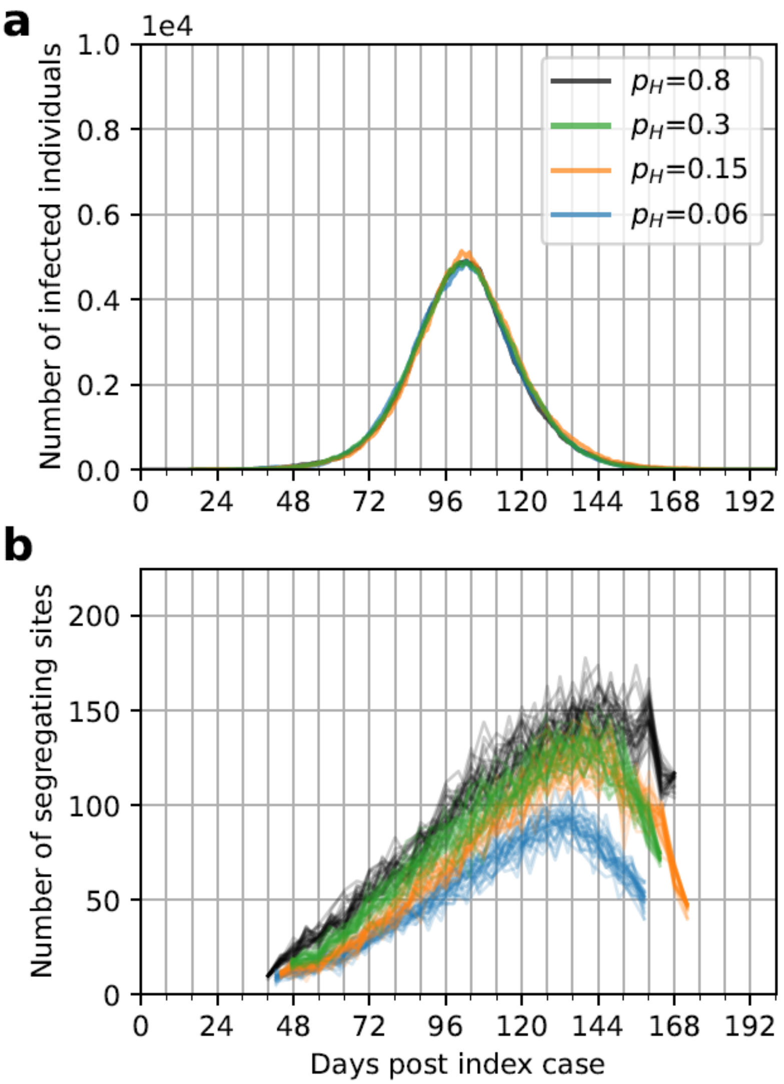

Figure S2. Segregating site trajectories under different levels of transmission heterogeneity. (a) Simulated dynamics of infected individuals (*I*) under an SEIR model with an R_0 of 1.6 and incorporating various levels of transmission heterogeneity compared to those of the original R_0 = 1.6 simulation without transmission heterogeneity. Transmission heterogeneity simulations shown are all shifted in time to align their epidemic peaks with the simulation without transmission heterogeneity (black line; p_H = 0.8). The transmission heterogeneity simulations considered span from low levels of transmission heterogeneity (p_H = 0.3), to intermediate levels of transmission heterogeneity (p_H = 0.15), to high levels of transmission heterogeneity (p_H = 0.06). (b) Segregating site trajectories for the simulations shown in (a). All simulations are densely sampled (40 sequences sampled per 4-day time window).

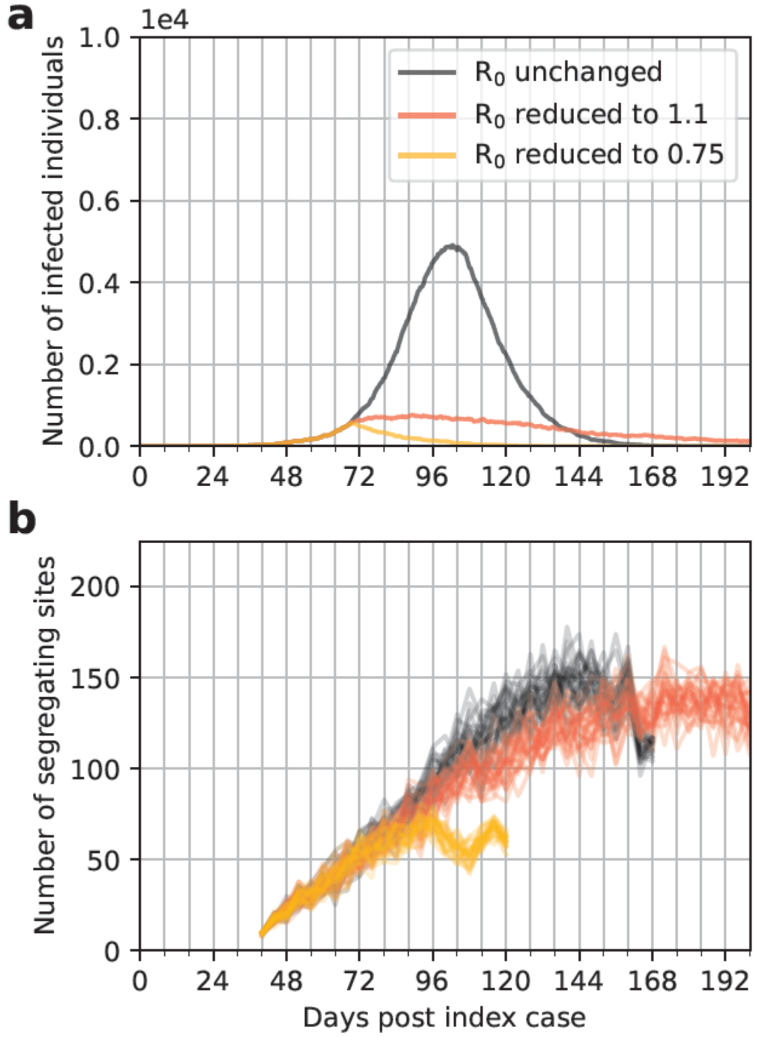

Figure S3. Segregating site trajectories under transmission reduction scenarios implemented at a later time point of the simulated epidemic. (a) Simulated dynamics of infected individuals (*I*) under an SEIR model. Changes in R_0 occurred when the number of infected individuals reached 1000. The simulation in red has R_0 decreasing to 1.1. The simulation in yellow has R_0 decreasing to 0.75. The simulation in black has R_0 remaining at 1.6. (b) Segregating site trajectories for the three simulations shown in (a). All simulations are densely sampled (40 sequences sampled per 4-day time window).

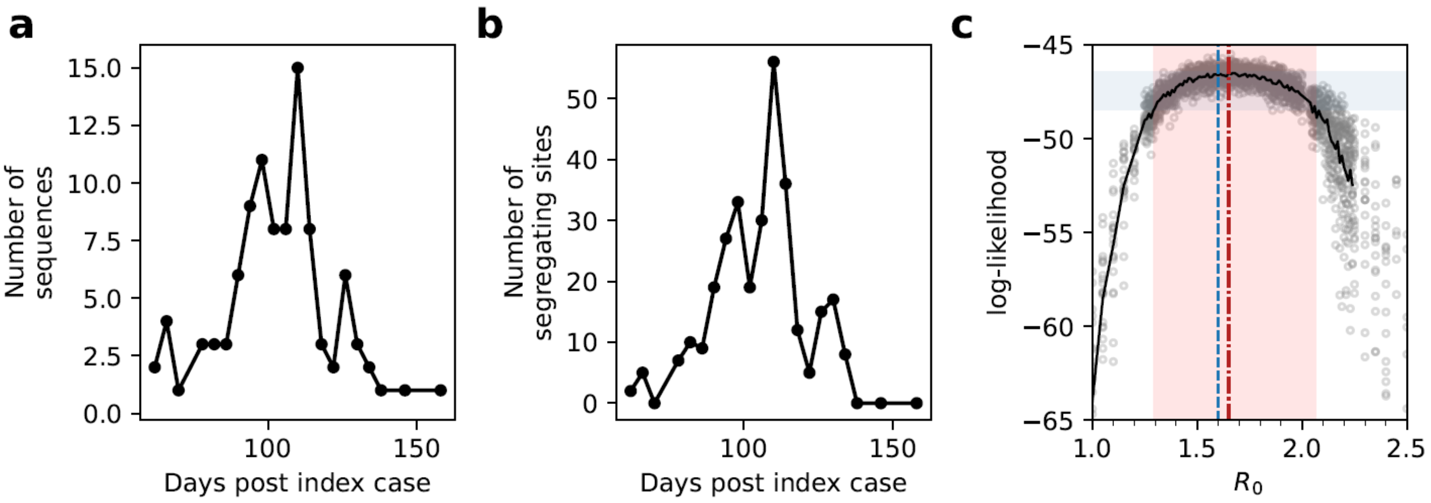

Figure S4. Epidemiological inference on a simulated trajectory of segregating sites, with lower sampling effort than in Figure 2. (a) The number of sampled sequences over time, by time window. Sampling was done in proportion to the number of individuals recovering in a time window. In all, 100 sequences were sampled over the course of the simulated epidemic. (b) Segregating site trajectory from the set of sampled sequences. (c) Estimation of R_0 using SMC. The maximum likelihood estimate for R_0 was 1.65 [95% CI = 1.30 to 2.06].

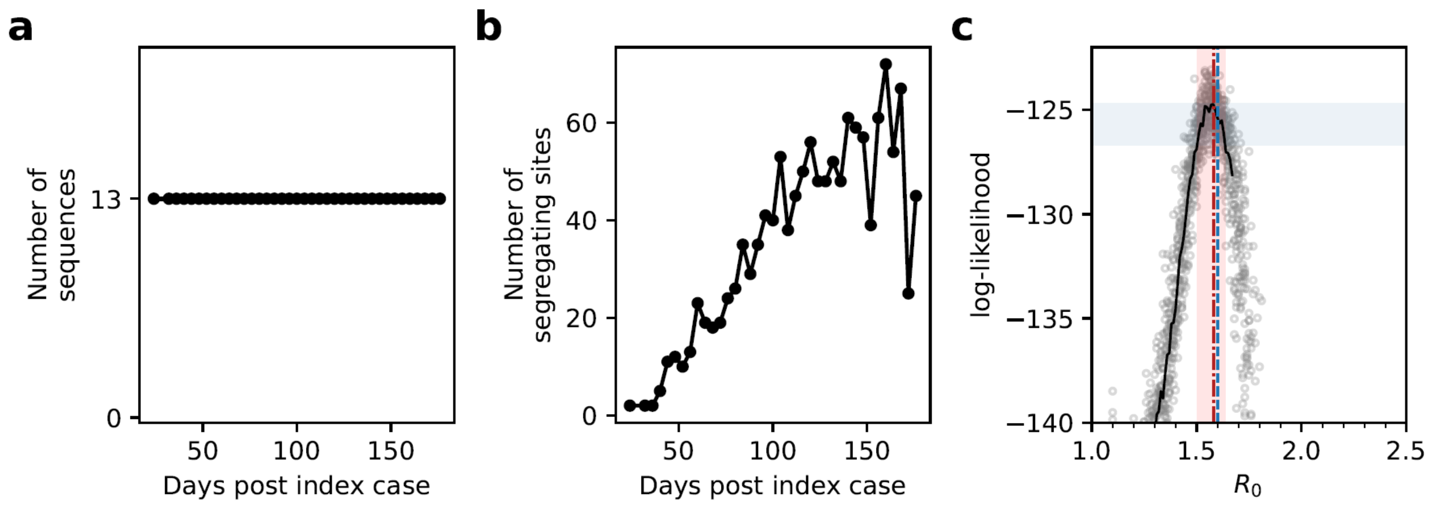

Figure S5. Epidemiological inference on a simulated trajectory of segregating sites, with uniform rather than proportional sampling. (a) The number of sampled sequences. Uniform sampling was performed by sampling 13 sequences per 4-day time window. Time windows with fewer than 13 sequences available were not included in the analysis. As such, here, only 494 sequences were used for inference. (b) Simulated segregating site trajectory from the sampled sequences. (c) Estimation of R_0 using SMC. The estimate for R_0 was 1.58 [95% CI = 1.51 to 1.62].

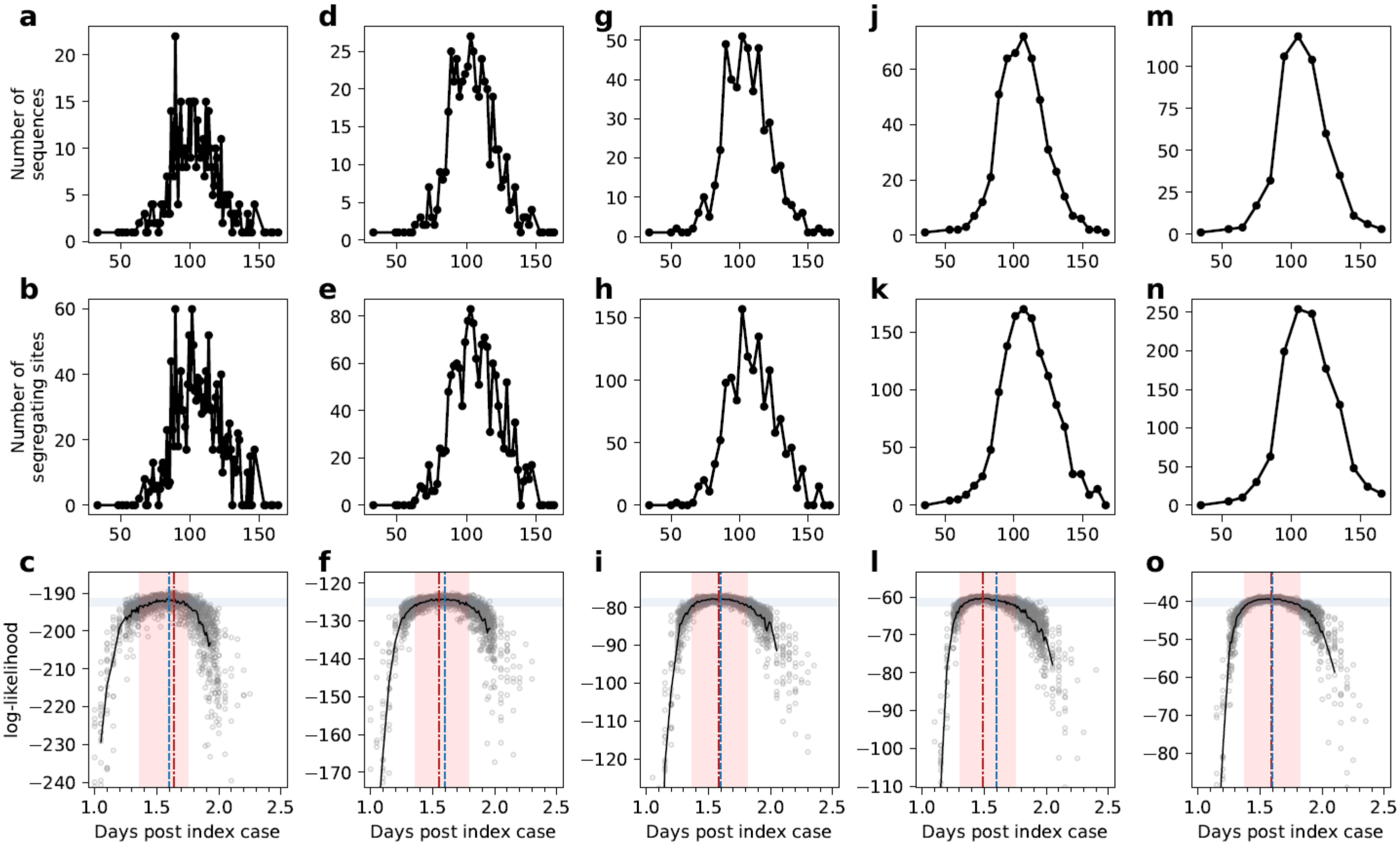

Figure S6. Epidemiological inference on the same set of sampled sequences as in Figure 2, binned at different time window lengths. For all time window lengths, the same set of 500 proportionally-sampled sequences were used. Columns show results by time window length: 1, 2, 4, 6, and 10 days, respectively. Top row (panels a,d,g,j,m): The number of sampled sequences over time, binned by time window. Middle row (panels b,e,h,k,n): Segregating site trajectories from the set of binned sequences. Bottom row (panels c,f,i,l,o): Estimation of R_0 using SMC. Panel c: the estimate for R_0 was 1.64 [95% CI = 1.36 to 1.75]. Panel f: the estimate for R_0 was 1.55 [95% CI = 1.36 to 1.79]. Panel i (reproducing the results shown in Figure 2): the estimate for R_0 was 1.58 [95% CI =1.37 to 1.81]. Panel l: the estimate for R_0 was 1.49 [95% CI = 1.31 to 1.75]. Panel o: the estimate for R_0 was 1.59 [95% CI = 1.38 to 1.82].

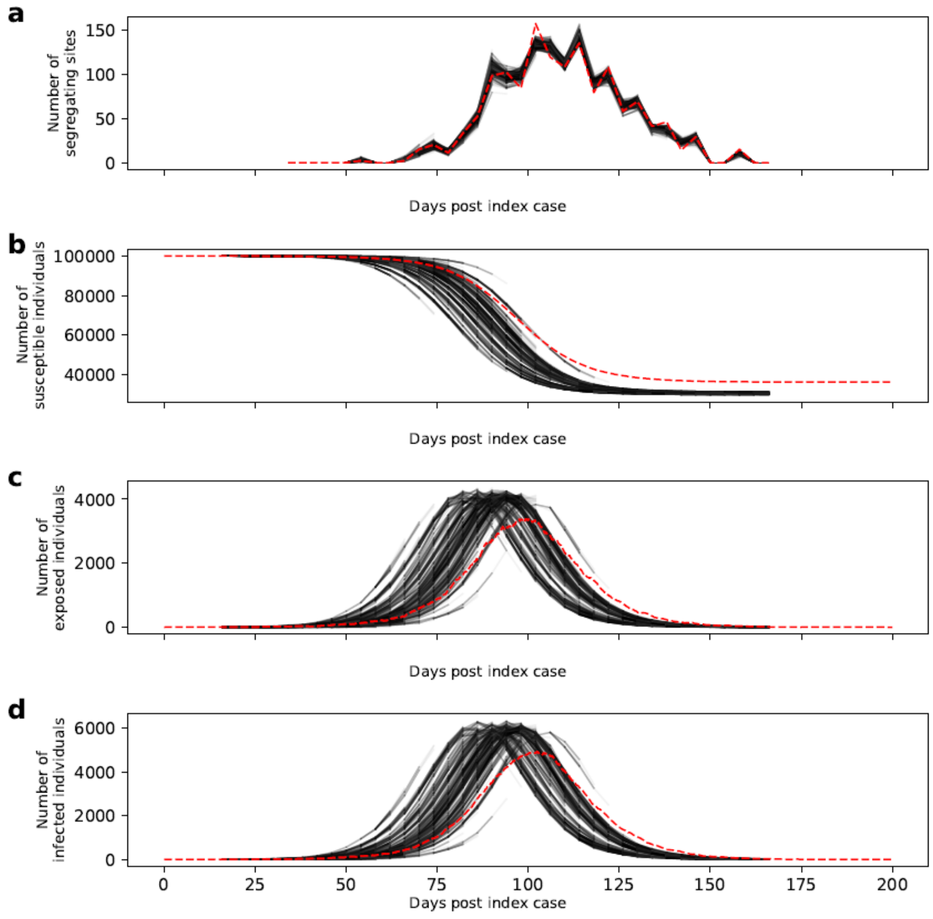

Figure S7. Resampling of particles during SMC allows for loss of low-weight particles with latent state variables that deviate from true ones. (a) Simulated trajectory of the number of segregating sites (dashed red), alongside reconstructed trajectories of the number of segregating sites (black lines). Gray lines show reconstructed segregating site trajectories from particles that were randomly sampled throughout the SMC procedure. (b) Simulated dynamics of susceptible individuals (dashed red), alongside reconstructed dynamics of susceptible individuals (black lines). Gray lines show reconstructed susceptible dynamics from particles that were randomly sampled throughout the SMC procedure. (c) Simulated dynamics of exposed individuals (dashed red), alongside reconstructed dynamics of exposed individuals (black lines). Gray lines show reconstructed dynamics of exposed individuals from particles that were randomly sampled throughout the SMC procedure. (d) Simulated dynamics of infected individuals (dashed red), alongside reconstructed dynamics of infected individuals (black lines). Gray lines show reconstructed dynamics of infected individuals from particles that were randomly sampled throughout the SMC procedure. Reconstructed dynamics from randomly sampled particles show state variables spanning from the sampled time point to the previous time point only. SMC simulations were run with R_0 = 1.7 and t_0 = 16, corresponding to the parameter combination with the highest mean log-likelihood value (see Figure 3a).

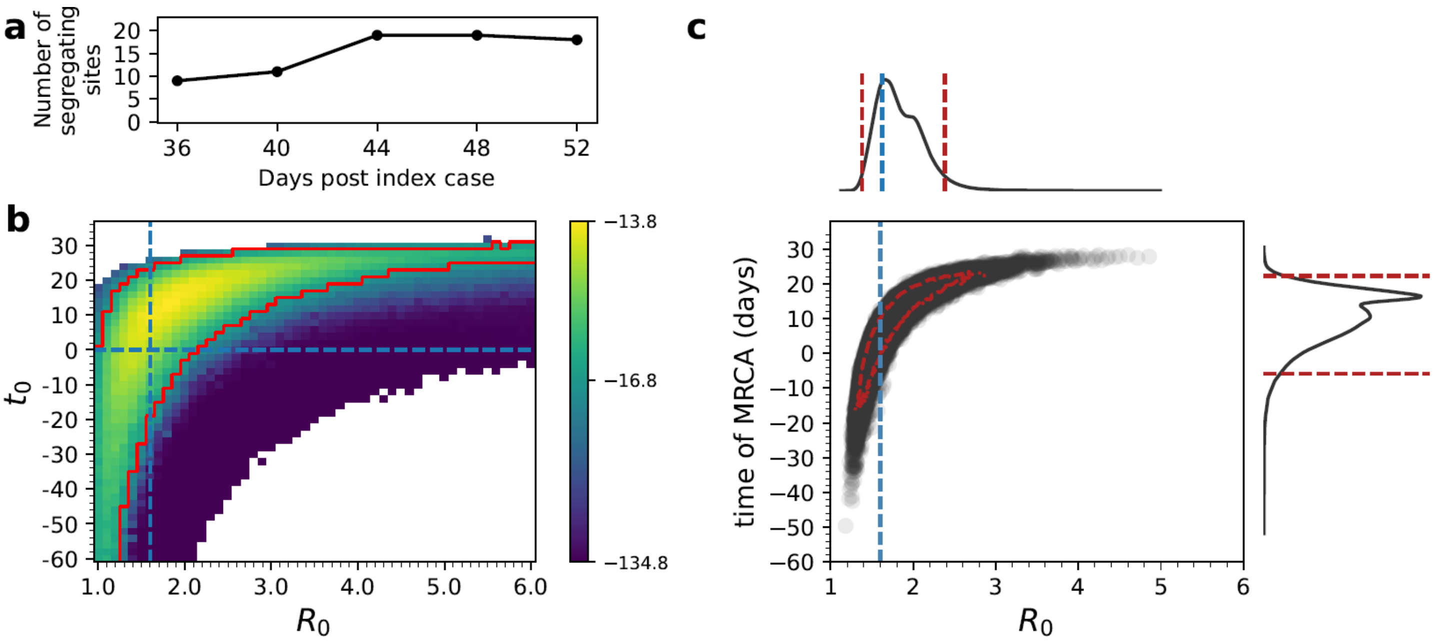

Figure S8. Joint estimation of the basic reproduction number (R_0) and the timing of the index case (t_0) using early samples from the short, μ = 0.4 simulation, with comparison against PhyDyn. (a) Simulated trajectory of the number of segregating sites using early sequences. Sequences were binned into 4-day windows, with 10 individuals sampled from each time window. (b) The log-likelihood surface based on a segregating site trajectory shown in panel (a). As in Figure 3a, the log-likelihood value shown in each cell is the mean log-likelihood value calculated from 20 SMC simulations and the 95% CI boundary shown in red contains sets of parameter combinations that fall within 2.966 log-likelihood units of the maximum log-likelihood. Blank cells had mean log-likelihood values of negative infinity. (c) Joint density plot for R_0 and the time of the most recent common ancestor (tMRCA), as estimated using PhyDyn [6] on the same set of 50 sampled sequences. Dashed red line in the joint density plot shows the 95% HPD interval of the joint density. The simulation was parameterized with a per genome, per transmission mutation rate of μ = 0.4.

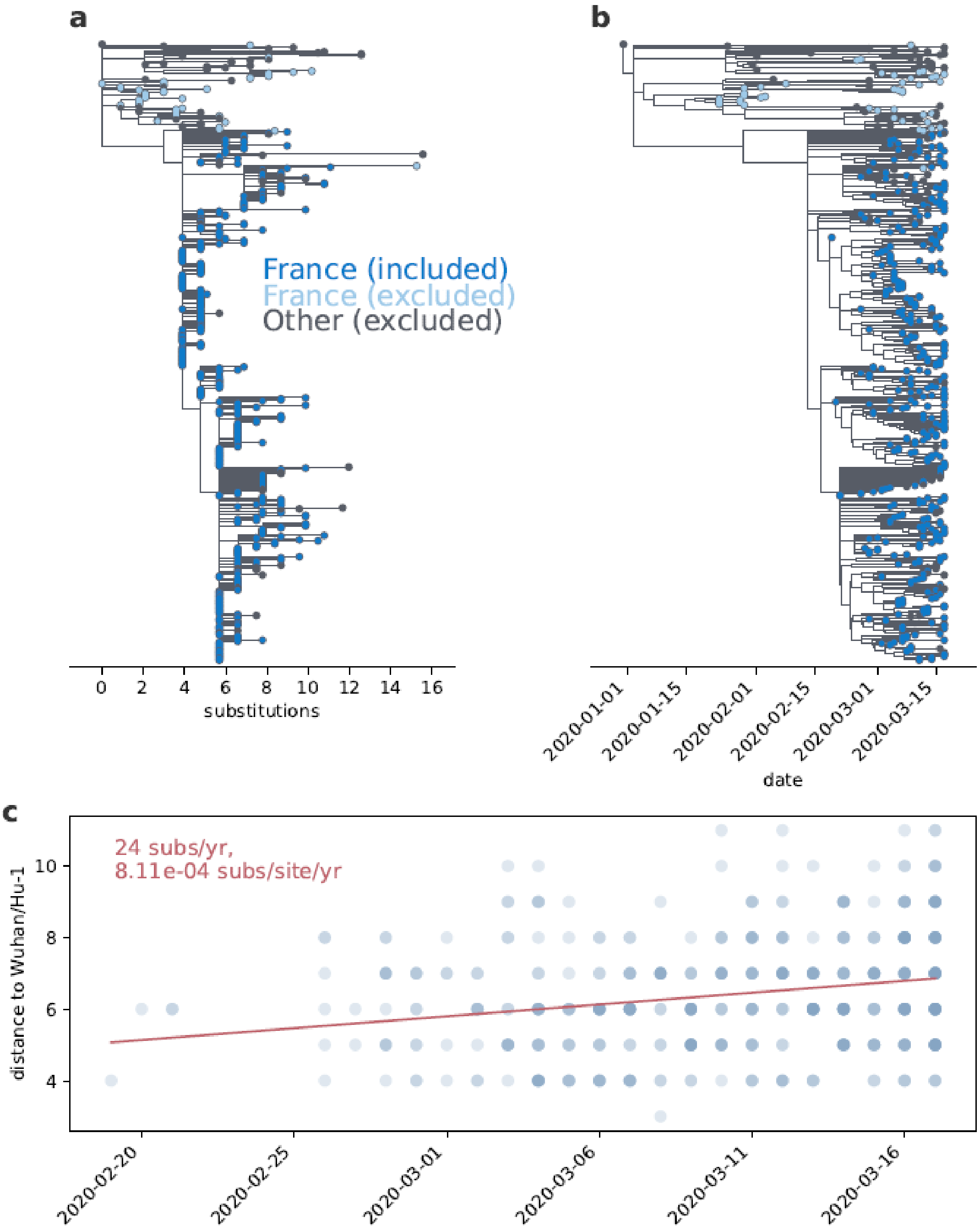

Figure S9. Inferred phylogenies for the sequences sampled from France, January 23-March 17, 2020. (a) Divergence tree, showing the number of nucleotide substitutions from Wuhan/Hu-1. Sequences from France are colored in blue, with dark blue coloring indicating sequences that were included in our single-lineage analysis and light blue coloring indicating sequences that were excluded from our analysis. Tips colored in gray denote genetically similar sequences sampled from outside of France during this time period. (b) Time-aligned maximum likelihood phylogeny, with coloring of sequences as in panel (a). (c) Plot showing genetic distances between sequences in the focal (dark blue) clade and the reference sequence Wuhan/Hu-1. A linear fit to these data yields a substitution rate of 8.11×10^{-4} substitutions per site per year, comparable to other reported substitution rates inferred for SARS-CoV-2 [1].

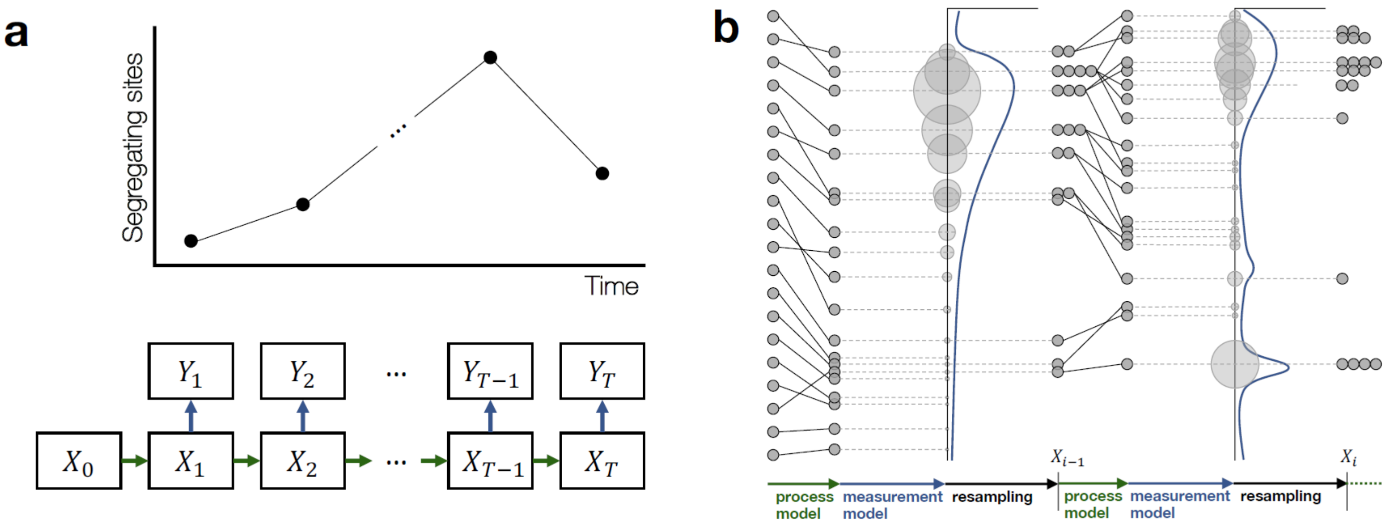

Figure S10. Depiction of the segregating sites inference approach using particle filtering. (a) Panel showing an observed segregating sites trajectory (top) and a schematic of a state-space model (bottom). A segregating site trajectory is obtained from available sequence data by first binning viral samples into consecutive, non-overlapping time windows according to their collection dates. The trajectory is then calculated by counting the number of polymorphic sites in the set of viral sequences in each time window. In a state-space model, the process model simulates underlying dynamics of latent variables over time. Here, the process model comprises the boxes labeled X_i (with i = 0, 1, …T-1, T) and the arrows between these boxes. The measurement model (depicted by the arrows between X_i and Y_i) relates the underlying state variables to the observed data Y_i. The observed data are the number of segregating sites over the time windows. (b) The particle filtering algorithm starts with a number of particles (shown as gray circles aligned in the first column), each initialized with initial state X_0. During a time window, the process model of each particle is simulated forward, arriving at a latent state X_1 at the end of the first time window. This is depicted by the black lines connecting the columns of particles. The measurement model is then used to calculate the weight of each particle (represented by the size of the light gray circles), which is defined as the probability of observing a given number of segregating sites for the time window i (s_i) based on each particle's simulated dynamics. We used Poisson distribution with rate parameter λ = mean s_i^{sim} (see Figure S11). Based on their weights, particles are sampled with replacement to generate a new set of particles for the next window. This resampling is shown using dotted lines that horizontally connect light gray circles to the gray-colored particles. This process continues until the last time window. Overall likelihoods of a given model parameterization are calculated by averaging the weights of the particles during each observation time window and then multiplying these average weights across time points.

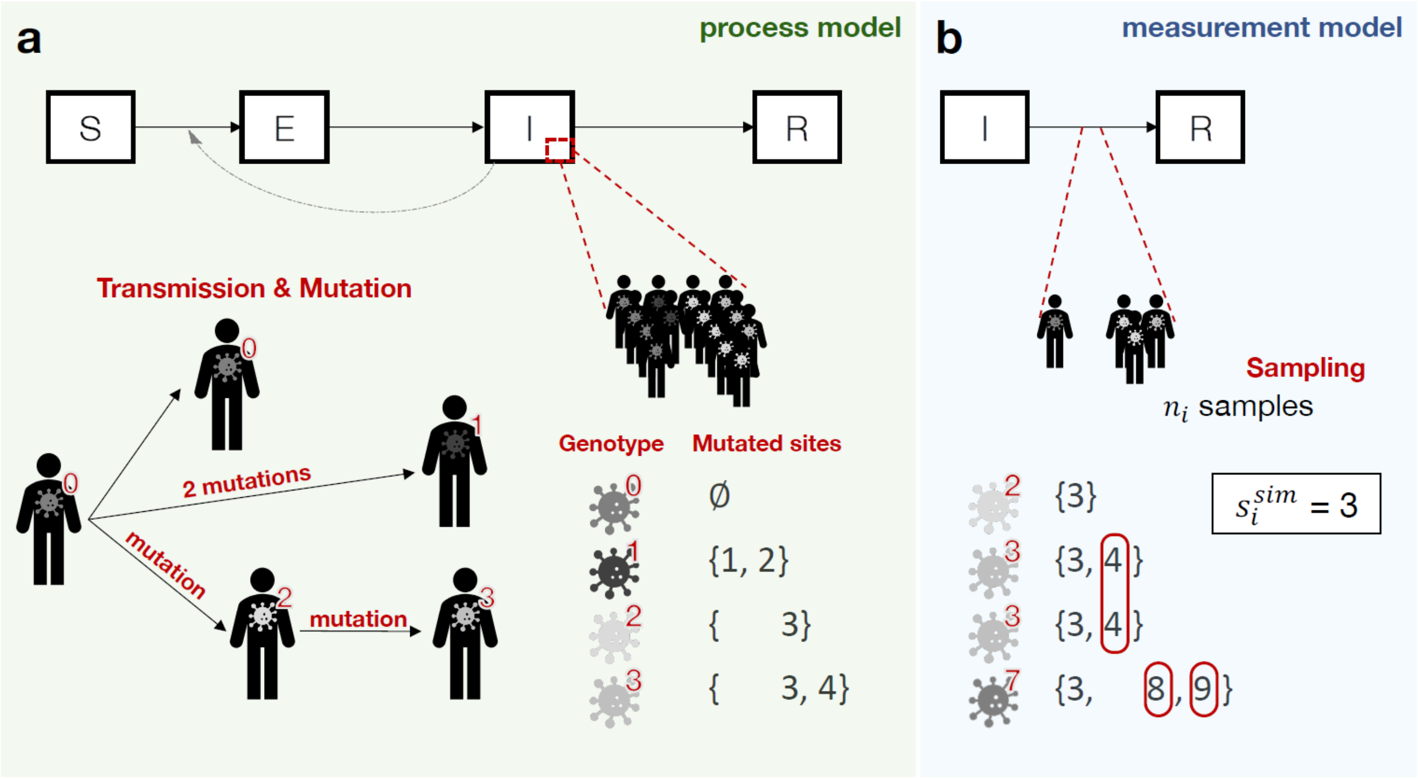

Figure S11. Depiction of the state space model. (a) Depiction of an epidemiological SEIR process model. The latent variables *X* in this process model are {*S*, *E*, *I*, and *R*}. Exposed and infected individuals are categorized by genotype, each of which has a unique set of mutations. Genotypes are shown as red-colored integers. A viral genotype of a donor is inherited by a recipient unless one or more mutations occur during the transmission event (bottom left). The occurrence of one or more mutations at transmission results in the recipient being infected with a new genotype, with this new genotype harboring the new mutation(s) as well as inheriting the existing set of mutations from the donor genotype. New mutations are numbered chronologically upwards from the current maximum mutation number. (b) Depiction of the measurement model. In addition to simulating the epidemiological dynamics specified by the process model, we keep track of the number of individuals of each genotype that have recovered during a given time window. In time window *i*, we then randomly sample n_i of these recovering individuals, where n_i denotes the number of viral samples that are binned in window *i* in the empirical data set. We calculate the number of segregating sites in this simulated sample of n_i sequences. Here, with $n_i = 4$, the number of segregating sites is $s_i^{sim} = 3$. These correspond to mutations 4, 8, and 9, because, for each of these sites, not all four sampled individuals carry the mutation. We repeat this process *k* times, with *k* 'grabs' of $n_i = 4$ recovering individuals and then calculate the mean number of segregating sites for that time window across these *k* grabs. Y_i in the state space model is given by this mean number of segregating sites in this time window *i*.

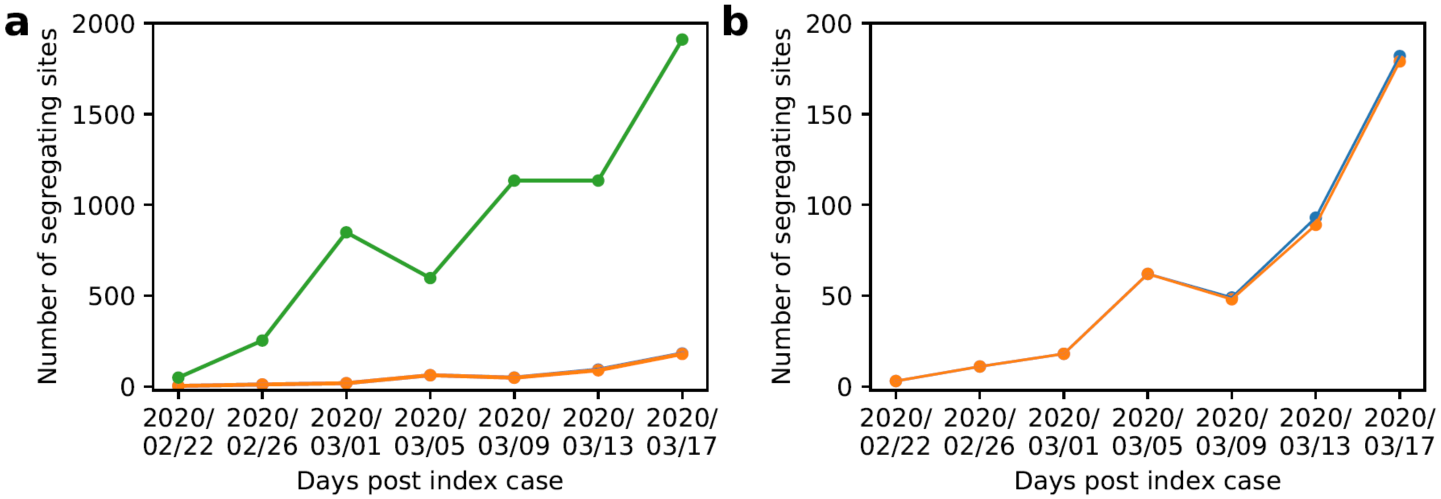

Figure S12. The effect of ambiguous nucleotides on the segregating site trajectory for France. (a) Segregating site trajectories calculated under different ambiguous nucleotide assumptions. The blue line shows the segregating site trajectory under the permissive assumption described in the main text (e.g., an *R* nucleotide matches both an *A* nucleotide and a *G* nucleotide). Under this assumption, an *N* nucleotide cannot increase the number of segregating sites observed in a time window, and an *R* nucleotide can only do so if the site at which it is found otherwise carries only *T*, *C*, and/or *Y* nucleotides. The green segregating site trajectory assumes that all ambiguous sites are mutations. Under this assumption, an *N* nucleotide increases the number of segregating sites observed in a time window unless that site already has all 4 nucleotides present, and an *R* nucleotide increases the number of segregating sites unless both an *A* and a *G* nucleotide are already present at that site. The orange segregating site trajectory assumes that *N* nucleotides match existing genetic variation but that other ambiguous nucleotides are considered as mutations. (b) Segregating site trajectories, as in panel (a), showing only the ambiguous nucleotide assumptions that correspond to the orange and blue lines in panel (a).

Table S1. Transmission pairs used to estimate the per-genome, per-transmission event mutation rate μ. Accession numbers of the consensus sequences from the donor and the recipient of the transmission pair are provided.

Study	Donor	Recipient	# SNPs	Study	Donor	Recipient	# SNPs
Popa et al. 2020	EPI_ISL_419656	EPI_ISL_437993	0	Braun et al. 2021	EPI_ISL_484813	EPI_ISL_484807	0
Popa et al. 2020	EPI_ISL_419656	EPI_ISL_437994	0	Braun et al. 2021	EPI_ISL_484919	EPI_ISL_484926	1
Popa et al. 2020	EPI_ISL_437994	EPI_ISL_437995	0	Braun et al. 2021	EPI_ISL_484919	EPI_ISL_484950	0
Popa et al. 2020	EPI_ISL_437994	EPI_ISL_438017	0	Braun et al. 2021	EPI_ISL_484926	EPI_ISL_484950	1
Popa et al. 2020	EPI_ISL_437994	EPI_ISL_438003	0	Braun et al. 2021	EPI_ISL_484921	EPI_ISL_484961	0
Popa et al. 2020	EPI_ISL_437994	EPI_ISL_438005	0	Braun et al. 2021	EPI_ISL_484921	EPI_ISL_484818	0
Popa et al. 2020	EPI_ISL_437994	EPI_ISL_437998	0	Braun et al. 2021	EPI_ISL_484961	EPI_ISL_484818	0
Popa et al. 2020	EPI_ISL_437994	EPI_ISL_583869	0	Braun et al. 2021	EPI_ISL_484952	EPI_ISL_484911	0
Popa et al. 2020	EPI_ISL_438005	EPI_ISL_438013	0	Braun et al. 2021	EPI_ISL_484973	EPI_ISL_484977	0
Popa et al. 2020	EPI_ISL_438005	EPI_ISL_438014	0	Braun et al. 2021	EPI_ISL_484976	EPI_ISL_495484	0
Popa et al. 2020	EPI_ISL_437998	EPI_ISL_438008	1	Braun et al. 2021	EPI_ISL_495461	EPI_ISL_509895	0
Popa et al. 2020	EPI_ISL_438008	EPI_ISL_438007	1	Braun et al. 2021	EPI_ISL_509876	EPI_ISL_509982	0
Popa et al. 2020	EPI_ISL_583869	EPI_ISL_438011	0	Braun et al. 2021	EPI_ISL_509876	EPI_ISL_509991	0
Popa et al. 2020	EPI_ISL_583869	EPI_ISL_583870	0	Braun et al. 2021	EPI_ISL_509876	EPI_ISL_509986	0
Popa et al. 2020	EPI_ISL_583869	EPI_ISL_438019	0	Braun et al. 2021	EPI_ISL_509982	EPI_ISL_509991	0
Popa et al. 2020	EPI_ISL_583869	EPI_ISL_438016	0	Braun et al. 2021	EPI_ISL_509982	EPI_ISL_509986	0
Popa et al. 2020	EPI_ISL_583869	EPI_ISL_583880	0	Braun et al. 2021	EPI_ISL_509991	EPI_ISL_509986	0
Popa et al. 2020	EPI_ISL_438016	EPI_ISL_438022	0	Braun et al. 2021	EPI_ISL_509897	EPI_ISL_509878	0
Popa et al. 2020	EPI_ISL_438022	EPI_ISL_438038	1	Braun et al. 2021	EPI_ISL_509897	EPI_ISL_509866	2
Popa et al. 2020	EPI_ISL_583870	EPI_ISL_438020	1	Braun et al. 2021	EPI_ISL_509878	EPI_ISL_509866	2
Popa et al. 2020	EPI_ISL_583870	EPI_ISL_438018	0	Braun et al. 2021	EPI_ISL_428254	EPI_ISL_428256	0
Popa et al. 2020	EPI_ISL_583870	EPI_ISL_583871	1	Braun et al. 2021	EPI_ISL_436627	EPI_ISL_436628	0
Popa et al. 2020	EPI_ISL_583870	EPI_ISL_475770	1	Braun et al. 2021	EPI_ISL_425176	EPI_ISL_427427	0
Popa et al. 2020	EPI_ISL_583871	EPI_ISL_583876	0	James et al. 2020	EPI_ISL_467433	EPI_ISL_467467	2
Popa et al. 2020	EPI_ISL_583871	EPI_ISL_583877	0	James et al. 2020	EPI_ISL_467433	EPI_ISL_467435	1
Popa et al. 2020	EPI_ISL_583871	EPI_ISL_583872	0	James et al. 2020	EPI_ISL_467433	EPI_ISL_467458	1
Popa et al. 2020	EPI_ISL_583872	EPI_ISL_583875	0	James et al. 2020	EPI_ISL_467446	EPI_ISL_467468	0
Popa et al. 2020	EPI_ISL_583872	EPI_ISL_583878	0	James et al. 2020	EPI_ISL_467446	EPI_ISL_467451	0
Popa et al. 2020	EPI_ISL_583875	EPI_ISL_438052	1	James et al. 2020	EPI_ISL_467444	EPI_ISL_467466	1
Popa et al. 2020	EPI_ISL_583875	EPI_ISL_438053	0	James et al. 2020	EPI_ISL_467444	EPI_ISL_467456	1
Popa et al. 2020	EPI_ISL_583875	EPI_ISL_438051	0	James et al. 2020	EPI_ISL_467444	EPI_ISL_467455	1

Popa et al. 2020	EPI_ISL_438051	EPI_ISL_438085	0
Popa et al. 2020	EPI_ISL_475770	EPI_ISL_583881	1
Popa et al. 2020	EPI_ISL_583880	EPI_ISL_438025	0
Popa et al. 2020	EPI_ISL_438039	EPI_ISL_438063	0
Popa et al. 2020	EPI_ISL_438100	EPI_ISL_438098	1
Popa et al. 2020	EPI_ISL_438035	EPI_ISL_438034	0
Popa et al. 2020	EPI_ISL_438035	EPI_ISL_438036	0
Popa et al. 2020	EPI_ISL_438035	EPI_ISL_438037	0
Braun et al. 2021	EPI_ISL_421299	EPI_ISL_421306	0
Braun et al. 2021	EPI_ISL_421323	EPI_ISL_421290	0
Braun et al. 2021	EPI_ISL_421327	EPI_ISL_421319	0
Braun et al. 2021	EPI_ISL_421328	EPI_ISL_421325	0
Braun et al. 2021	EPI_ISL_421332	EPI_ISL_421287	0
James et al. 2020	EPI_ISL_467444	EPI_ISL_467432	1
James et al. 2020	EPI_ISL_467444	EPI_ISL_467433	0
James et al. 2020	EPI_ISL_467444	EPI_ISL_467442	0
Lythgoe et al. 2021	NA	NA	0
Lythgoe et al. 2021	NA	NA	2
Lythgoe et al. 2021	NA	NA	1
Lythgoe et al. 2021	NA	NA	0
Lythgoe et al. 2021	NA	NA	0
Lythgoe et al. 2021	NA	NA	0
Lythgoe et al. 2021	NA	NA	1
Lythgoe et al. 2021	NA	NA	2
Lythgoe et al. 2021	NA	NA	0

Sequence data downloaded from GISAID.

Data Availability

GISAID Identifier: EPI_SET_230123mt
doi: 10.55876/gis8.230123mt

All genome sequences and associated metadata in this dataset are published in GISAID's EpiCoV database. To view the contributors of each individual sequence with details such as accession number, Virus name, Collection date, Originating Lab and Submitting Lab and the list of Authors, visit 10.55876/gis8.230123mt

Data Snapshot

- EPI_SET_230123mt is composed of 13,963 individual genome sequences.
- The collection dates range from 2019-10-22 to 2020-11-02;
- Data were collected in 103 countries and territories;
- All sequences in this dataset are compared relative to hCoV-19/Wuhan/WIV04/2019 (WIV04), the official reference sequence employed by GISAID (EPI_ISL_402124). Learn more at https://gisaid.org/WIV04.

Supplementary References

1. Duchene, S. *et al.* Temporal signal and the phylodynamic threshold of SARS-CoV-2. *Virus Evolution* **6**, veaa061 (2020).
2. Volz, E. M. & Siveroni, I. Bayesian phylodynamic inference with complex models. *PLoS Comput Biol* **14**, e1006546 (2018).
3. Le Vu, S. *et al.* Prevalence of SARS-CoV-2 antibodies in France: results from nationwide serological surveillance. *Nat Commun* **12**, 3025 (2021).
