# Supplementary material for: Epidemiological inference for emerging viruses using segregating sites
Source: Nat Commun. 2023 May 29;14:3105. doi: 10.1038/s41467-023-38809-7 (PMC10226718; doi:10.1038/s41467-023-38809-7)
Supplement: Supplementary file 3 — Reporting Summary [file 41467_2023_38809_MOESM3_ESM.pdf]

nature portfolio

Corresponding author(s): Koelle

Last updated by author(s): Mar 29, 2023

Reporting Summary

Nature Portfolio wishes to improve the reproducibility of the work that we publish. This form provides structure for consistency and transparency in reporting. For further information on Nature Portfolio policies, see our Editorial Policies and the Editorial Policy Checklist.

Statistics

For all statistical analyses, confirm that the following items are present in the figure legend, table legend, main text, or Methods section.

n/a	Confirmed	
☐	☒	The exact sample size (n) for each experimental group/condition, given as a discrete number and unit of measurement
☐	☒	A statement on whether measurements were taken from distinct samples or whether the same sample was measured repeatedly
☒	☐	The statistical test(s) used AND whether they are one- or two-sided<br>*Only common tests should be described solely by name; describe more complex techniques in the Methods section.*
☒	☐	A description of all covariates tested
☒	☐	A description of any assumptions or corrections, such as tests of normality and adjustment for multiple comparisons
☐	☒	A full description of the statistical parameters including central tendency (e.g. means) or other basic estimates (e.g. regression coefficient) AND variation (e.g. standard deviation) or associated estimates of uncertainty (e.g. confidence intervals)
☒	☐	For null hypothesis testing, the test statistic (e.g. F, t, r) with confidence intervals, effect sizes, degrees of freedom and P value noted<br>*Give P values as exact values whenever suitable.*
☐	☒	For Bayesian analysis, information on the choice of priors and Markov chain Monte Carlo settings
☐	☒	For hierarchical and complex designs, identification of the appropriate level for tests and full reporting of outcomes
☒	☐	Estimates of effect sizes (e.g. Cohen's d, Pearson's r), indicating how they were calculated

Our web collection on statistics for biologists contains articles on many of the points above.

Software and code

Policy information about availability of computer code

Data collection	No software or code was used for data collection.
Data analysis	PhyDyn v1.3.8, implemented in BEAST2, was used. The code for the segregating sites inference approach developed in this manuscript, and all code for the generation of figures, is .provided on our GitHub site: https://github.com/koellelab/segregating-sites. This url is provided in the manuscript under Code Availability.

For manuscripts utilizing custom algorithms or software that are central to the research but not yet described in published literature, software must be made available to editors and reviewers. We strongly encourage code deposition in a community repository (e.g. GitHub). See the Nature Portfolio guidelines for submitting code & software for further information.

Data

Policy information about availability of data

All manuscripts must include a data availability statement. This statement should provide the following information, where applicable:

- Accession codes, unique identifiers, or web links for publicly available datasets
- A description of any restrictions on data availability
- For clinical datasets or third party data, please ensure that the statement adheres to our policy

Simulated data are provided at the GitHub site (https://github.com/koellelab/segregating-sites). Table S2 provides GISAID information on the SARS-CoV-2 sequence data from France that were used in our analyses. Accession numbers for the transmission pairs used to infer the mutation rate are provided in Table S1.

Field-specific reporting

Please select the one below that is the best fit for your research. If you are not sure, read the appropriate sections before making your selection.

☐ Life sciences ☐ Behavioural & social sciences ☒ Ecological, evolutionary & environmental sciences

For a reference copy of the document with all sections, see nature.com/documents/nr-reporting-summary-flat.pdf

Ecological, evolutionary & environmental sciences study design

All studies must disclose on these points even when the disclosure is negative.

Study description	This manuscript presents a new statistical inference approach to infer epidemiological model parameters from viral sequence data. We test our approach on simulated data, as well as apply our method to existing SARS-CoV-2 sequence data from France.
Research sample	Full-genome SARS-CoV-2 sequence data, available on GISAID.
Sampling strategy	We queried GISAID for full-genome SARS-CoV-2 sequences from France collected on or before March 17, 2020.
Data collection	Data were downloaded from GISAID by coauthor Michael A. Martin and stored locally on his Mac.
Timing and spatial scale	Sequences collected in France on or before March 17, 2020 were considered in the analysis. This is because March 17, 2020 commenced the SARS-CoV-2 lock-down in France, which changed contact rates following this date. We considered viral spread specifically in France to compare our results to those from an epidemiological analysis for this country.
Data exclusions	Viral sequences not falling within the major France clade were excluded, as described in detail in the manuscript. This exclusion was implemented because our inference approach only considers a single viral clade.
Reproducibility	All findings are reproducible with the code provided on GitHub.
Randomization	There were no aspects of this study that involved randomization as randomization was not applicable for this type of study. his study involves time series analysis, not an experimental study.
Blinding	There were no aspects of this study that involved blinding as blinding was not applicable for this type of study. This study involves time series analysis, not an experimental study.

Did the study involve field work? ☐ Yes ☒ No

Reporting for specific materials, systems and methods

We require information from authors about some types of materials, experimental systems and methods used in many studies. Here, indicate whether each material, system or method listed is relevant to your study. If you are not sure if a list item applies to your research, read the appropriate section before selecting a response.

Materials & experimental systems

n/a	Involved in the study	
☒	☐	Antibodies
☒	☐	Eukaryotic cell lines
☒	☐	Palaeontology and archaeology
☒	☐	Animals and other organisms
☒	☐	Human research participants
☒	☐	Clinical data
☒	☐	Dual use research of concern

Methods

n/a	Involved in the study	
☒	☐	ChIP-seq
☒	☐	Flow cytometry
☒	☐	MRI-based neuroimaging
